# Determinants of Telehealth Adoption Among Older Adults: Cross-Sectional Survey Study

**DOI:** 10.2196/60936

**Published:** 2025-03-24

**Authors:** Siow-Hooi Tan, Yee-Yann Yap, Siow-Kian Tan, Chee-Kuan Wong

**Affiliations:** 1 Faculty of Management, Multimedia University Cyberjaya Malaysia; 2 School of Economics and Management, Xiamen University Malaysia Sepang Malaysia; 3 Department of Medicine, Faculty of Medicine, Universiti Malaya Kuala Lumpur Malaysia

**Keywords:** telehealth services adoption, survey, questionnaire, telehealth, older adult population, subjective well-being, transition cost, technology acceptance model, importance-performance map analysis, IPMA

## Abstract

**Background:**

The aging population and the accompanying rise in chronic diseases have intensified the need to study the adoption of telehealth services. However, the success of telehealth services depends not only on their ease and usefulness but also on addressing broader concerns. Despite being a substantial user group in traditional health services, older adults may encounter barriers to adopting telehealth services. Increasing the adoption of telehealth among the older adult population is crucial for enhancing their access to care and managing the challenges of aging effectively.

**Objective:**

We aimed to explore factors influencing the adoption of telehealth services among older adults in Malaysia, going beyond the conventional framework by incorporating transition cost and subjective well-being as additional constructs.

**Methods:**

A cross-sectional survey was conducted among 119 adults aged ≥60 years in Malaysia, using 39 survey items adapted from existing studies. Data analysis was performed using partial least squares structural equation modeling, with both the measurement model and structural model being evaluated. To determine the predictive relevance of the model, PLSpredict was applied. In addition, importance-performance map analysis was conducted to further expand on the structural model results by assessing the performance of each variable.

**Results:**

Of the 119 participants, 52 (43.7%) were women and 67 (56.3%) were men. The study found that subjective well-being (β=0.448; *P*<.001) was the most significant factor, followed by attitude (β=0.242; *P*<.001), transition cost (β=−0.163; *P*<.001), and perceived usefulness (β=0.100, *P*=.02) in influencing telehealth service intention. Furthermore, perceived ease of use (β=0.271; *P*<.001), availability (β=0.323; *P*<.001), subjective well-being (β=0.261; *P*<.001), and trust (β=0.156, *P*=.004) positively influenced perceived usefulness, while inertia (β=0.024, *P*=.22) did not. In addition, availability (β=0.420; *P*<.001) and subjective well-being (β=0.260; *P*<.001) were positively related to perceived ease of use, with inertia (β=−0.246; *P*<.001) having a negative impact. The importance-performance map analysis results showed that subjective well-being (importance=0.532) was the most crucial factor for older adult users, while availability (importance=70.735) had the highest performance in telehealth services.

**Conclusions:**

This research underscores the importance of catering to the subjective well-being of older adults and optimizing the availability of telehealth services to encourage adoption, ultimately advancing health care accessibility and quality for this vulnerable demographic.

## Introduction

### Background

Telehealth refers to the delivery of long-distance clinical health care services by health care professionals using electronic information and telecommunications technologies. Due to the growth of the internet and communication infrastructure, telehealth has gradually developed into a practical and secure way for patients to obtain reliable information and medical consultation [1]. Using telehealth has several advantages, such as eliminating the need for direct patient–health care provider interaction during regular treatment. Telehealth can also provide remote care, which can reduce the need for medical center resources and increase the accessibility of care.

Previous telehealth studies have developed various concepts to address how telehealth could fulfill the needs of older adults, such as in the context of chronic disease management, enhancing independent living and improving their overall well-being [2]. Telehealth can be useful for older adults with chronic diseases to monitor their conditions at home. For instance, with the use of telehealth, older adult patients can prevent unnecessary hospitalization and still ensure they receive emergency treatment in a cost-efficient manner [3,4]. In addition, Chou et al [5] found that telehealth can improve the well-being of older adults by enhancing their quality of life. Furthermore, telehealth can promote independent living at home among older adults [6].

Older adults could benefit from telehealth as they are the fragile groups who may need this service sooner or later. Nevertheless, they are also the group most concerned about technology. The literature provides evidence that older adults can receive several advantages with the use of telehealth services, such as health monitoring and care, disease prevention, improved quality of life, and independent living. Despite all the advantages, the adoption of telehealth technology may be challenging for older adults because they are slower and more resistant to adopting new technology as they tend to be more traditional, cautious, risk-averse, and suspicious toward innovations [7]. There is a lack of knowledge about what factors individuals will consider when accepting telehealth [8]. The market response indicates that acceptance of technology by older adults is a complex problem that is impacted by a variety of factors rather than just the technology’s performance or cost [9].

In the context of Malaysia, telehealth is becoming increasingly important in the health care system, especially with the older adult population expected to exceed 15% by 2030 [10]. This demographic shift will place added pressure on the health care system, particularly due to a rise in chronic diseases [11]. Malaysia’s health care system offers accessible services but faces a workforce shortage to meet growing demands. With 2.4 physicians per 1000 people—fewer than those in Singapore, Japan, and Australia—Malaysia faces an aging population and staffing shortages leading to overcrowded public hospitals and strained health care capacity [11]. This has drastically burdened the health care system in Malaysia.

In response, telehealth offers a vital solution to address the growing imbalance between health care supply and demand as health care needs continue to rise [12]. The Malaysian government has been actively exploring technological solutions and launching telehealth initiatives to address the rising health care needs of its aging population. For instance, Malaysia’s Ministry of Health initiated a teleconsultation at public hospitals to improve health care access and reduce congestion. Despite these efforts and the recent surge in telehealth-related studies, there remains a scarcity of research to investigate telehealth adoption in emerging economies, especially from the older adults’ perspective [11,13]. Investigating telehealth adoption by older adults across different countries is essential, as varying levels of technological development and cultural contexts substantially influence their attitudes and behaviors [13,14].

Previous studies on technology adoption among older adults span various cultural contexts, revealing distinct factors influencing their behavior. In Canada, a study by Ahmed et al [15] found that >half of older adults adopted new technology for online social interactions. Despite having the knowledge to stay connected, they faced challenges like limited access and motivation. In a cross-cultural survey by Elimelech et al [16], older adults in Israel, France, and Spain exhibited different perceptions of technology use, emphasizing the need for culturally tailored adaptations. In Australia, Catapan et al [17] reported that patients had a high level of confidence and trust in the use of telehealth. Meanwhile, in China, Lin et al [18] showed that telehealth’s ease of use and usefulness played a substantial role in affecting its adoption. In a developed country such as Singapore, Zhang et al [19] found that telehealth effectively supports the health-seeking behavior of older adults, challenging the belief that they resist technology and lack proficiency. Similarly, Haimi and Sergienko [20] found that telehealth uses among older adults in Israel remained elevated after the COVID-19 pandemic, indicating their ability to effectively learn and use digital health services.

However, in Malaysia, older adults may have different perceptions of telehealth. According to Ting et al [21], many older adults still prefer face-to-face interactions due to a cultural preference for personal consultations. Reservations about the impersonal nature of telehealth remain a substantial obstacle. However, the behavior of older adults toward telehealth in emerging economies, such as Malaysia, remains underexplored. There is a need for research focused on telehealth adoption within the Malaysian context to gain better grasp of this context.

### Theory

Researchers have introduced various theoretical models to explain consumer behavior in the context of technology adoption. Well-established models for predicting technology acceptance among consumers include the theory of planned behavior by Ajzen [22], the technology acceptance model (TAM) by Davis et al [23], TAM2 by Venkatesh and Davis [24], the unified theory of acceptance and use of technology by Venkatesh et al [25], and TAM3 by Venkatesh and Bala [26], among others.

A growing body of research highlights the reliability and effectiveness of the TAM in explaining technology adoption. TAM, introduced by Davis et al [23], uses the concepts of perceived usefulness and perceived ease of use to elucidate how technological factors influence a consumer’s intent to adopt a specific technology. For the adoption of health-related technologies, the TAM and the unified theory of acceptance and use of technology have emerged as 2 prominent models, as demonstrated in studies by Harst et al [27], Heinsch et al [28], Rouidi et al [29], and Lin et al [18]. In the context of the aging population, a recent systematic literature review by Yap et al [30] confirmed that the TAM is the most widely used theory for explaining technology adoption among older adults.

Despite the widespread use of the TAM in the literature, previous studies argued that solely using the fundamental TAM to identify the consumer’s technology adoption is insufficient [31,32]. Similarly, Attié and Meyer-Waarden [33] criticized that functional and utilitarian benefits, such as perceived ease of use, are insufficient to explain technology acceptance. Therefore, it can be found that previous studies have frequently extended the original TAM with additional variables or other theories to better reflect the technology’s acceptance. For instance, Zhou et al [34] extended the original TAM by incorporating perceptions of medical service quality and information quality into the model for predicting telehealth acceptance among older adults. Telehealth acceptance, in turn, is influenced by older adults’ perceptions of telehealth and their current behavioral intentions toward telehealth services. In addition, Rho et al [8] extended the TAM with perceived incentives, self-efficacy, and accessibility of patients’ medical records, while Klingberg et al [35] included image, self-efficacy, voluntariness, compatibility, and anxiety in the TAM fundamental framework to enhance the explanatory power of the TAM. Moreover, integration of theories and perspectives has been performed as well, such as in the study by Tsai et al [36] that integrated the TAM with the status quo bias and technology anxiety concept to explain the telehealth intention. Therefore, it is suggested that there is a need for research to expand the TAM by including additional variables to provide a more comprehensive explanation and understanding regarding the telehealth intention of older adults.

### Objectives

The objectives of this study are as follows:

- To investigate the coexistence and possible effects of TAM constructs, transition cost, and subjective well-being on telehealth service adoption among older adults
- To examine how inertia, availability, subjective well-being, and trust relate to the TAM’s key antecedents

## Methods

### Research Model and Hypothesis Development

#### Overview

Telehealth is an effective and advanced alternative method for delivering health care services. In the context of telehealth adoption, the fundamental TAM might be insufficient to explain the telehealth adoption among older adults. In particular, the older adult population might often hold negative opinions about technology’s inaccuracies that would influence their intention to use it [37]. Considering the evolving landscape of telehealth adoption among older adults in Malaysia and the limited existing literature, this study contributes to the expansion of the TAM. It does so by introducing and exploring a set of key factors aimed at comprehending the older adult population’s willingness to embrace telehealth. In addition to attitude and perceived usefulness, this study proposes 2 additional constructs: transition cost and subjective well-being, to evaluate older adults’ intention to use telehealth.

First, subjective well-being is included to address critiques of the traditional TAM. Recent studies [11,32,33] argue that the TAM’s focus solely on utilitarian benefits is insufficient to fully explain consumer technology adoption. On the basis of transformative consumer studies and the uses and gratification theory [38], it is evident that beyond utilitarian benefits like usefulness and ease of use, consumers also seek affective elements, such as well-being when adopting a new technology [33]. The literature suggests enhancing the TAM by incorporating affective elements like subjective well-being to more accurately predict technology adoption [11]. Nevertheless, subjective well-being has received minimal attention in telehealth adoption studies, particularly from the perspective of the older adult population. Therefore, research proposes that subjective well-being be considered as one of the predictors of older adults’ intention to use telehealth. If older adults perceive that telehealth can enhance their well-being, they are more likely to adopt it, as people naturally seek experiences that improve their overall quality of life [39,40].

The inclusion of transition cost as an extension to the TAM is grounded in the status quo bias theory, which posits that individuals tend to prefer maintaining their current routines over adopting change [41]. This is especially relevant for older adults, who often find transitions, such as shifting to telehealth, burdensome due to the perceived effort, time, and disruption of familiar health care practices like face-to-face consultations. Many older adults grew up in a time when technological innovations were not widespread, leading them to develop long-standing routines that provide comfort and predictability [42]. As a result, this study proposes that transition costs are particularly impactful for this group, as they are more likely to resist switching to telehealth in favor of maintaining their familiar health care practices.

**Figure 1:**
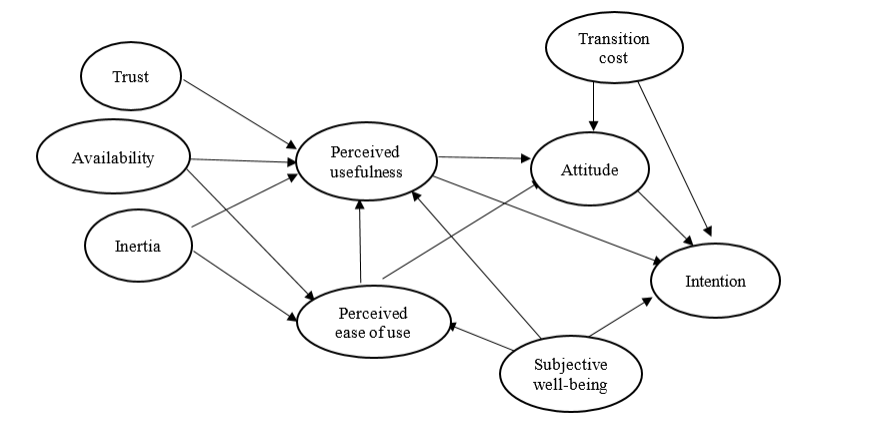
Research model.

Furthermore, the research incorporates availability, trust, inertia, and subjective well-being as factors influencing the TAM constructs. Subsequent sections of this study will provide a detailed analysis of the significance of these constructs within the unique context of this research. Figure 1 shows the research model of this study. The proposed hypotheses based on the developed research model are discussed in subsequent sections.

#### Attitude

Attitude is an evaluation of effect, which refers to a person’s positive or negative feelings regarding performing the respective behavior [43]. The impact of attitude on behavioral intention is a significant relationship in the theory of reasoned action, the theory of planned behavior, and the TAM. The connection between one’s attitude and their intention signifies that individuals are more inclined to embrace technology if they hold a favorable perception of it, as noted by Davis et al [23]. The concept of attitude has played an important role in numerous studies seeking to gain deeper insights into consumers’ willingness to adopt health care technologies. Previous research has consistently confirmed that a positive attitude toward health care–related technologies among consumers has a substantial impact on their intent to use such technologies, as evidenced by studies conducted by Park et al [44], Papa et al [45], Rajak and Shaw [46], and Ahn and Park [47].

Furthermore, Tsai et al [36] unveiled that the older adults’ attitudes exert a positive influence on their intention to adopt telehealth. In addition, prior research has shown that attitude can serve as a mediator between beliefs and behavioral intent, as demonstrated by the work of Yang and Yoo [48]. Consequently, this study proposed the following hypothesis:

- Hypothesis 1: there is a positive association between attitude and the intention to adopt telehealth.

#### Perceived Ease of Use

Perceived ease of use is defined as the degree to which an older adult perceives that using telehealth technology would be free of effort. Perceived ease of use is one of the key constructs in the TAM. According to the TAM, perceived ease of use is related to attitudes and perceived usefulness of new technologies, which also influences intention [24]. Previous research has found that perceived ease of use influences consumers’ attitudes and perceived usefulness toward new technologies [31,36,46,47]. For instance, Lazaro et al [49] confirmed that perceived ease of use positively affects the older adults’ perceived usefulness and their attitude toward wearable health care technology. Hence, we proposed the following hypothesis:

- Hypothesis 2a: there is a positive association between perceived ease of use and perceived usefulness.
- Hypothesis 2b: there is a positive association between perceived ease of use and attitude.

#### Perceived Usefulness

Perceived usefulness is defined as the degree to which an individual feels that using a certain technology will improve their job performance [23]. Researchers have supported perceived usefulness as a crucial factor that predicts various types of technology adoption among older adults, such as the internet [50], social networking sites [51], health monitoring wearable technologies [52], telehealth [53], and automation technology [54]. Besides, previous studies revealed that perceived usefulness was highly relevant in predicting telehealth acceptance among the older adult population [34,55]. In addition, perceived usefulness influences consumers’ attitudes toward new technologies [36,46,47]. Therefore, the following hypothesis is proposed:

- Hypothesis 3a: there is a positive association between perceived usefulness and attitude.
- Hypothesis 3b: there is a positive association between perceived usefulness and intention to adopt telehealth.

#### Transition Cost

According to Kim and Kankanhalli [56], transition costs refer to the user’s perceived disutility that they incur when switching from the status quo to a new technology. When older adults consider using new technology, such as telehealth, the transition costs involved are essential. Transition costs that are uncertain might become a barrier and negatively influence a person’s attitude [57]. In addition, Hsieh [58] revealed that the transition cost that might be incurred when using health-related technology is one of the key concerns for technology adoption. An individual is likely to continue and remain with an existing system if the transition costs involved, such as effort and time, to learn to use a new technology, are deemed to be high. In the context of telehealth adoption, Tsai et al [36] claimed that transition costs negatively affect older adults’ attitudes and hence affect their telehealth adoption. Therefore, if the transition cost to use a new technology is high, older adults will have a negative attitude toward it and not intend to use it. On the basis of the discussions, the following hypothesis is proposed:

- Hypothesis 4a: there is a negative association between transition cost and attitude.
- Hypothesis 4b: there is a negative association between transition cost and intention to adopt telehealth.

#### Subjective Well-Being

Subjective well-being refers to an individual’s perception of an experience positively by using affective reactions and cognitive judgment instead of objective facts [59]. When adopting new technology, people always seek pleasurable experiences that enhance their well-being [39,40]. Previous studies have revealed that well-being influences consumer technology adoption [60]. Al-Jabri and Sohail [61] revealed that technology’s characteristics contribute to a person’s well-being. Similarly, well-being could act as a determinant that influences technology use [40]. Wu and Lu [62] have highlighted that positive emotions become a motivator for technology adoption when a person uses technology. In addition, findings show that well-being toward technology positively influences an individual’s perceived ease of use, usefulness, and intention across all consumer adoption phrases [33,63,64]. Consumers will develop positive emotions toward technology when they perceive that using it will enhance their well-being. Therefore, positive emotions will positively influence the perceived benefits, such as perceived usefulness and ease of using the technology [33].

Therefore, this study proposes that older adults perceive that telehealth will enhance their well-being, which consequently will influence their perceived ease of use, usefulness, and intention to use telehealth. Therefore, we hypothesize the following:

- Hypothesis 5a: there is a positive association between well-being and perceived ease of use.
- Hypothesis 5b: there is a positive association between well-being and perceived usefulness.
- Hypothesis 5c: there is a positive association between well-being and intention to adopt telehealth.

#### Inertia

Inertia refers to the degree of a person’s willingness to continue using traditional physical products despite knowing that better options are available [41]. Even when better alternatives or switching incentives are available, consumers remain attached to and steadfast in their use of existing technologies [65]. Hence, the greater an individual’s attachment toward a thing that he or she is familiar with, the less inclined they are to explore new experiences. In addition, Bem [66] and Petty and Cacioppo [67] claimed that people usually depend on their prior behavior and hence fail to recognize a new technology’s advantages. In line with this assumption, older adults always seek to maintain their existing internal and external structures when making adaptive decisions, as they would prefer to continue to engage in similar activities or behaviors as they did throughout their previous experiences [68]. The older adult population grew up in an era when technological innovation was not commonly used. As a result of their early experiences, they might have long been accustomed to receiving health care services physically at hospitals or clinics. Older adults might perceive telehealth as not useful and not easy to operate as they prefer to continue to engage in the behavior they are more familiar with to minimize feelings of anxiety. It is hypothesized that inertia will negatively influence the older adults’ behavioral perceptions of a new technology and hence create lower inclinations to use new technologies. Individuals who have high inertia tend to reduce the variety of technologies that are available to them and rely on prior behavior to influence their perceptions and intentions [36]. Therefore, the following hypothesis is proposed:

- Hypothesis 6a: there is a negative association between inertia and perceived ease of use.
- Hypothesis 6b: there is a negative association between inertia and perceived usefulness.

#### Availability

On the basis of the study by Venkatesh [69], availability refers to the extent to which consumers perceive that they can obtain a technological service or product without barriers, along with the presence of organizational support to help them overcome any challenges in using the technology. A person’s control belief about the availability of organizational resources and support structures to enable technology use is related to facilitating conditions [26]. Many previous studies have proved the positive impact of facilitating conditions on technology adoption [70,71]. In the telehealth adoption context, the determinant associated with external control might involve the availability of manufacturer’s assistance, where the firms provide consumers assistance to overcome the difficulties of using a new technology. Telehealth technologies can make medical resources available to health care professionals, caregivers, and older adults at any time and from any location, allowing a considerable improvement in patient health care. Wu et al [72] revealed that availability positively influences the perceived usefulness of the telehealth care technology. In addition, previous studies also provided support on the impact of availability in the context of telehealth adoption [73,74]. Similarly, a recent study by Tsai et al [36] revealed that availability is an important predictor in determining perceived ease of use and usefulness of telehealth among the older adult population. According to the existing evidence, this study hypothesizes that availability would increase the older adults’ perceived ease of use and usefulness of telehealth.

- Hypothesis 7a: there is a positive association between availability and perceived ease of use.
- Hypothesis 7b: there is a positive association between availability and perceived usefulness.

#### Trust

Trust is evidently a crucial determinant in health care adoption. On the basis of the study by Gefen et al [75], trust refers to a sense of confidence in the trustworthiness and integrity of the other party. When it comes to technological adoption, trust is essential, especially when the technology is relatively new and might involve risks and uncertainties for the older adult population. Despite trust not being included in the original TAM, it has been incorporated into several of the study contexts. Many existing studies demonstrate that trust has a strong positive influence on technology adoption [45,76]. Previous studies also revealed that trust plays an important role in predicting adoption of health-related technologies (eg, telehealth) [76,77].

In the context of this study, telehealth can be a difficult and complex task, especially for older adults, as its use requires a good understanding of devices to communicate effectively with the health care providers [78,79]. Hence, people might lack trust in using telehealth due to the risks incurred, including unclear regulatory authority in place to deal with issues like confidentiality, misconduct, and liability in telehealth. Older adults, who used to obtain services physically, might have trust issues with telehealth’s ability to replace in-person consultations and physical health assessments. Particularly in the IT setting, Li et al [80] revealed that trust is crucial, as people must overcome the perceived risk before technology adoption. Previous research has established the importance of trust in determining health-related technology adoption [46]. For instance, Catapan et al [17], Chew et al [81], and Orrange et al [82] showed that trust substantially affects telehealth adoption. Hence, this study hypothesizes that trust would increase older adults’ perceived usefulness of telehealth.

- Hypothesis 8: there is a positive association between trust and perceived usefulness.

### Research Instrument Development

The measurement scales were adjusted in accordance with existing literature and tailored to suit our research context. The questionnaire was divided into 2 sections. The first part consisted of the 11 constructs used in this study: attitude, availability, transition cost, perceived ease of use, perceived usefulness, inertia, trust, subjective well-being, and intention to adopt. The second part comprised a survey focusing on demographic characteristics, such as gender and ethnicity. An overview of the instrument is provided in Multimedia Appendix 1 [32,36,83,84].

The scales for attitude, availability, transition cost, perceived ease of use, perceived usefulness, and inertia were taken from the study of Tsai et al [36] and Zhang and Zaman [83]. The measures for subjective well-being were based on the work of Yap et al [32]. Trust and intention were measured according to the work of Wu et al [84]. In total, 39 items were assessed using a 5-point Likert scale, which ranged from *completely disagree* (score=1) to *completely agree* (score=5).

To ensure the questionnaire’s reliability, a pilot survey was carried out involving 10 older adults. This pretest, which involved contacting 10 older adults before conducting the web-based survey, was conducted to validate the instrument. On the basis of the feedback received, minor adjustments were made to the questionnaire to improve its effectiveness.

### Research Sample and Data Collection Procedure

This study focused on the factors influencing the intention to adopt telehealth among adults aged ≥60 years in Malaysia. This demographic was chosen as research participants due to their increased susceptibility to chronic diseases, as outlined by Tsai et al [36]. Chronic diseases can affect individuals of all ages, but the risk escalates as people advance in age, justifying the selection of this population for our research.

In our data collection process, we used a survey approach to investigate the intent of older adults to adopt telehealth services. Recognizing the challenges in directly reaching this demographic, we engaged students as intermediaries to connect with their family members who were older. This method combines elements of convenience and snowball sampling, as students were encouraged to distribute surveys within their social networks, primarily targeting their older family members. We encouraged student intermediaries to recruit participants from diverse geographic regions and socioeconomic backgrounds to enhance sample diversity where possible.

The rationale behind using student intermediaries was 2-fold: first, it provided a practical and effective means of reaching older adult participants, a group that may not be as digitally connected or comfortable with technology. The student intermediaries, who were trained to administer the survey, acted as trusted conduits, facilitating communication and engagement with older adult participants in a familiar, trustworthy, and less intimidating environment. This helped to overcome potential barriers related to accessibility and comprehension of the survey, ensuring that the older adult participants felt comfortable and supported throughout the process. Hence, it was essential for the student intermediaries to have a thorough understanding of both the study’s purpose and the questionnaire to effectively guide their older family members. To ensure this, students underwent a comprehensive briefing before the survey, equipping them with a solid grasp of the questionnaire and its objectives.

### Data Analysis

This study conducted data analysis using partial least squares structural equation modeling (PLS-SEM). We used SmartPLS 3.2.8 [85] for PLS-SEM, as it is well-suited for analyzing measurement and structural models without the need for normality assumptions. This is particularly useful because survey research data are often not normally distributed [86]. Furthermore, PLS-SEM offers a higher explanatory power compared to covariance-based structural equation modeling.

### Ethical Considerations

This study received formal approval from the Research Ethics Committee of Multimedia University (EA2882021). Informed written consent was obtained from all survey respondents before participation. Respondents were required to read the ethical statement at the top of the survey form and proceed only if they agreed to participate. All collected data are treated with the utmost confidentiality, ensuring anonymity and used solely for research purposes. Additionally, consent to publish was obtained from all participants.

## Results

### Respondent Characteristics

The survey was conducted from March 1, 2022, to August 31, 2022, resulting in 125 received samples, of which 119 were considered valid. While this approach offers advantages for reaching a challenging-to-access population, researchers should remain vigilant about potential biases arising from the familial and social connections of the student intermediaries. In the sample, 52 (43.7%) of the 119 respondents were women, and 67 (56.3%) were men. Additional demographic details are provided in Multimedia Appendix 2.

### Reliability and Validity Tests

The evaluation of the measurement model involved assessing reliability and validity. In Table 1, all variables exhibited factor loadings >0.7 [87], and Cronbach α exceeded 0.7, signifying a high level of reliability. Both the composite reliability and average variance extracted surpassed 0.7 and 0.5, respectively, indicating strong convergent validity [88]. In the second step of the analysis, we evaluated discriminant validity using the heterotrait-monotrait (HTMT) ratio criterion, as suggested by Henseler et al [89] and Franke and Sarstedt [90]. As indicated in Table 2, the results of the HTMT criterion demonstrate that all HTMT values fall below the threshold of 0.85 for the more stringent criterion. This indicated that the respondents recognized the distinctiveness of all the constructs. The combination of these 2 validity tests affirms that the measurement items exhibited both validity and reliability.

**Table 1:** Measurement model and cross-validated redundancy.

Constructs and items			Loadings		Composite reliability		Average variance extracted
**Attitude**					0.896		0.743
	ATT^a^1	0.833					
	ATT2	0.850					
	ATT3	0.900					
**Perceived ease of use**					0.963		0.897
	EOU^b^1	0.938					
	EOU2	0.954					
	EOU3	0.948					
**Perceived usefulness**					0.928		0.762
	USE^c^1	0.841					
	USE2	0.857					
	USE3	0.902					
	USE4	0.890					
**Transition cost**					0.953		0.872
	COST^d^1	0.920					
	COST2	0.939					
	COST3	0.942					
**Inertia**					0.889		0.728
	INE^e^1	0.889					
	INE2	0.811					
	INE3	0.858					
**Availability**					0.886		0.723
	AVAI^f^1	0.862					
	AVAI2	0.888					
	AVAI3	0.798					
**Trust**					0.900		0.751
	TRUST^g^1	0.886					
	TRUST2	0.808					
	TRSUT3	0.903					
**Subjective well-being**					0.959		0.885
	SWB^h^1	0.934					
	SWB2	0.948					
	SWB3	0.941					
**Intention**					0.932		0.821
	INT^i^1	0.911					
	INT2	0.885					
	INT3	0.923					

^a^ATT: attitude.

^b^EOU: perceived ease of use.

^c^USE: perceived usefulness.

^d^COST: transition cost.

^e^INE: inertia.

^f^AVA: availability.

^g^TRUST: trust.

^h^SWB: subjective well-being.

^i^INT: intention.

**Table 2:** Discriminant validity (heterotrait-monotrait 0.85 criterion).

	ATT^a^	EOU^b^	USE^c^	COST^d^	INE^e^	AVA^f^	TRUST^g^	SWB^h^	INT^i^
ATT	—^j^	—	—	—	—	—	—	—	—
EOU	0.677	—	—	—	—	—	—	—	—
USE	0.769	0.719	—	—	—	—	—	—	—
COST	0.549	0.623	0.498	—	—	—	—	—	—
INE	0.390	0.427	0.261	0.523	—	—	—	—	—
AVA	0.665	0.654	0.781	0.453	0.140	—	—	—	—
TRUST	0.688	0.530	0.683	0.389	0.210	0.527	—	—	—
SWB	0.755	0.577	0.715	0.170	0.374	0.545	0.753	—	—
INT	0.753	0.674	0.701	0.570	0.432	0.579	0.677	0.818	—

^a^ATT: attitude.

^b^EOU: perceived ease of use.

^c^USE: perceived usefulness.

^d^COST: transition cost.

^e^INE: inertia.

^f^AVA: availability.

^g^TRUST: trust.

^h^SWB: subjective well-being.

^i^INT: intention.

^j^Not applicable.

### Results of the Structural Model

Following the guidance of Hair et al [91], we reported the path coefficients, SEs, 1-tailed *t* test values, and *P* values using a bootstrapping procedure with 5000 resamples [92]. This large resample size ensures result stability, according to Hair et al [91] and Hair and Alamer [93]. In addition, Hahn and Ang [94] emphasized that *P* values alone were insufficient for hypothesis significance and recommended combining *P* values, effect sizes, and bias-corrected interval for a more comprehensive evaluation. Table 3 presents the evaluation of the hypotheses. Moreover, the results of bias-corrected interval, effect sizes, and variance inflation factor are presented in Multimedia Appendix 3.

**Table 3:** Hypothesis testing direct effects^a^.

Hypothesis	Relationship	Standard β	SD	*t* test value	*P* value
Hypothesis 1	Attitude → intention	0.242	0.058	4.168	<.001
Hypothesis 2a	Perceived ease of use → perceived usefulness	0.271	0.048	5.693	<.001
Hypothesis 2b	Perceived ease of use → attitude	0.209	0.056	3.734	<.001
Hypothesis 3a	Perceived usefulness → attitude	0.456	0.054	8.450	<.001
Hypothesis 3b	Perceived usefulness → intention	0.100	0.050	1.997	.02
Hypothesis 4a	Transition cost → attitude	−0.153	0.055	2.779	.003
Hypothesis 4b	Transition cost → intention	−0.163	0.037	4.357	<.001
Hypothesis 5a	Subjective well-being → perceived ease of use	0.260	0.053	4.927	<.001
Hypothesis 5b	Subjective well-being → perceived usefulness	0.261	0.057	4.531	<.001
Hypothesis 5c	Subjective well-being → intention	0.448	0.047	9.582	<.001
Hypothesis 6a	Inertia → perceived ease of use	−0.246	0.041	5.972	<.001
Hypothesis 6b	Inertia → perceived usefulness	0.024	0.034	0.778	.22
Hypothesis 7a	Availability → ease of use	0.420	0.048	8.725	<.001
Hypothesis 7b	Availability → perceived usefulness	0.323	0.047	6.818	<.001
Hypothesis 8	Trust → perceived usefulness	0.156	0.060	2.620	.004

^a^We used 95% CI with a bootstrapping of 5000.

First, we examined the influence of the 4 predictors on intention. Attitude (β=0.242; *P*<.001), perceived usefulness (β=0.100, *P*=.02), transition cost (β=−0.163; *P*<.001), and subjective well-being (β=0.448; *P*<.001) were all associated with intention. This means that hypotheses 1, 3b, 4b, and 5c were supported.

Second, the impact of the 3 predictors on attitude was investigated. Perceived ease of use (β=0.209; *P*<.001), perceived usefulness (β=0.456; *P*<.001), and transition cost (β=−0.153; *P*<.001) were linked to attitude, affirming support for hypotheses 2b, 3a, and 4a.

Third, perceived usefulness was assessed in relation to 5 predictors. Perceived ease of use (β=0.271; *P*<.001), availability (β=0.323; *P*<.001), subjective well-being (β=0.261; *P*<.001), and trust (β=0.156; *P*<.001) exhibited positive associations, while inertia (β=.024, *P*=.22) showed no significant relationship with perceived usefulness. Consequently, hypotheses 2a, 5b, 7b, and 8 were supported, while hypothesis 6b was not.

Finally, we examined the impact of availability (β=.420; *P*<.001) and subjective well-being (β=0.260; *P*<.001) on perceived ease of use, finding that both were positively related, while inertia (β=−0.246; *P*<.001) was negatively related. This supported hypotheses 5a, 6a, and 7a.

Consistent with the results of previous hypothesis testing, the bias-corrected 95% CIs for all hypotheses (except 6a) confirmed that they did not encompass 0, indicating support for these hypotheses (Multimedia Appendix 3). Furthermore, the variances for intention to adopt (*R*^2^=0.650), attitude (*R*^2^=0.502), perceived usefulness (*R*^2^=.654), and perceived ease of use (*R*^2^=0.475) were generally above 33%. This suggests the model possesses a moderate predictive capacity.

To assess the predictive relevance of the model, Shmueli et al [95] suggested the use of PLSpredict, a holdout sample-based method that produces case-level predictions at either the item or construct level. We used PLSpredict with a 10-fold procedure to assess predictive capability. As demonstrated in Table 4, all the errors associated with the PLS model were lower compared to the linear model, indicating the robust predictive power of our model. Therefore, we can confidently assert that our model exhibits strong predictive capacity.

**Table 4:** PLSpredict results.

Items for the construct intention	PLS^a^	LM^b^	PLS-LM
INT1^c^	0.734	0.735	−0.001
INT2	0.856	0.866	−0.010
INT3	0.767	0.769	−0.002

^a^PLS: partial least squares.

^b^LM: linear model.

^c^INT: intention.

### Results of the Importance-Performance Map Analysis

The importance-performance map analysis (IPMA) of older adults’ intention to adopt telehealth services aimed to expand upon the results of the structural model by evaluating the performance of each variable. As mentioned by Hair et al [91], areas requiring management attention are those with high importance but poor performance on a specific endogenous latent variable. In our research, we assessed the impact of latent exogenous factors on the endogenous variable (ie, intention to adopt) in terms of their significance and performance. The results of this analysis are presented in Table 5.

**Table 5:** Importance-performance map analysis.

Constructs	Performance	Importance
Attitude	63.245	0.241
Perceived ease of use	60.779	0.108
Perceived usefulness	64.078	0.210
Transition cost	52.758	0.200
Inertia	69.450	0.021
Availability	70.735	0.113
Trust	64.529	0.033
Subjective well-being	53.330	0.532

The IPMA results showed that the most important factor was subjective well-being (0.532), followed by attitude (0.241), perceived usefulness (0.210), transition cost (0.200), availability (0.113), perceived ease of use (0.108), trust (0.033), and inertia (0.021).

On the basis of performance, availability (70.735) was the highest, followed by inertia (69.45), trust (64.53), perceived useful (64.078), attitude (63.22), ease of use (60.779), subjective well-being (53.33), and transition cost (52.758). Therefore, it is evident that subjective well-being, attitude, transition cost, and perceived usefulness play crucial roles in influencing the intention of older adults to adopt telehealth, as these constructs exhibit relatively higher total effects (importance) compared to other factors in the model. However, the performance of well-being, attitude, transition cost, and usefulness were relatively lower compared to other factors like availability, inertia, and trust.

In summary, to enhance the intention to adopt telehealth among older adults, managerial efforts should be primarily focused on addressing and emphasizing subjective well-being, while continuing to support and maintain positive attitudes and perceived usefulness. On the other hand, perceived ease of use should be given lower priority.

## Discussion

### Principal Findings

This study aims to enhance the existing TAM for the adoption of telehealth services among older adults in the Malaysian context. Apart from attitude and perceived usefulness, this study proposed 2 additional constructs, namely subjective well-being and transition cost, for assessing the intention of older adults to use telehealth. Furthermore, this study introduced availability, trust, inertia, and subjective well-being as antecedents of TAM constructs. The study found that subjective well-being is the most important factor in telehealth adoption, followed by attitude, transition cost, and perceived usefulness. Perceived ease of use, perceived usefulness, and transition cost substantially affected attitude. Perceived ease of use, availability, subjective well-being, and trust positively influenced perceived usefulness, while inertia did not. In addition, availability and subjective well-being were positively related to perceived ease of use, with inertia having a negative impact. IPMA results showed that subjective well-being was the most crucial factor for older adult users, while availability had the highest performance in telehealth services.

As expected, this study found that attitude positively affects intention, which is in line with the existing health care–related studies, such as those by Park et al [44], Papa et al [45], Rajak and Shaw [46], and Ahn and Park [47]. As per the results, the key TAM construct, perceived ease of use, positively influences older adults’ perceived usefulness and attitude toward the telehealth system. These outcomes corroborate the findings of Rajak and Shaw [46] and Ahn and Park [47], which indicate that older adults will have a positive attitude toward telehealth and perceive it to be useful to them if telehealth is easy to use. On the other hand, perceived usefulness also positively influences older adults’ attitude and intention toward telehealth. Specifically, perceived usefulness has a significantly larger influence on attitudes regarding adopting telehealth than perceived ease of use (0.456 vs 0.209). The rationale behind these findings may be due to the relevance of using telehealth for managing older adults’ health. Hence, the usefulness of telehealth is the priority as compared to its ease of use. In other words, older adults are more likely to use telehealth services if they can provide useful features to them, such as improving their quality of life and offering better health care services.

Consistent with previous findings [36,57,58], these findings show that transition costs are driving forces that have a negative impact on the older adults’ attitude and intention to use telehealth. According to the findings, older adults do not intend to use telehealth and will continue using the traditional method of obtaining health care services if they believe the time and effort required to learn telehealth is too high.

Besides, inertia was found to negatively influence the perceived ease of use but had no impact on the perceived usefulness. The significance of inertia’s influence on perceived ease of use stems from the fact that ease of use is directly linked to how simple or user-friendly telehealth appears. When older adults experience inertia, they tend to resist adopting telehealth because of the additional effort required to learn and adapt to new technologies. This resistance (inertia) makes new systems seem more complex, thereby reducing the perceived ease of use of telehealth among older adults. However, the significance of inertia on perceived ease of use contradicts the findings of Tsai et al [36], where inertia had no effect on ease of use. This discrepancy may stem from the fact that the majority of respondents in the study by Tsai et al [36] were younger (>40 years) with higher digital literacy, while our study focuses on those aged ≥60 years. In contrast, the insignificant impact of inertia on perceived usefulness in this study is consistent with the findings of Tsai et al [36]. This is because perceived usefulness is more about the functional benefits the user gains from the technology. Hence, even if older adults perceive the system to be hard to use due to inertia, they might still acknowledge that the system provides value or could be beneficial in terms of performance and efficiency. This can explain why inertia does not affect perceived usefulness to the same extent.

Availability showed a positive influence on ease of use and perceived usefulness, which is consistent with existing studies, such as those by Chang et al [74] and Tsai et al [36]. However, these findings partially conflict with the findings of Wu et al [72], which revealed that availability only influences the perceived usefulness and has no impact on the perceived ease of use. This revealed different perspectives between patients and hospital professionals toward the importance of availability in determining telehealth adoption. From the older adult users’ perspective, the study’s findings revealed that the availability of assistance, such as providing responses to assist them in overcoming obstacles when using telehealth, is very important, which would affect their perceived ease of use and usefulness of telehealth.

This study revealed that well-being positively influences perceived ease of use, perceived usefulness, and intention toward telehealth. These outcomes are expected as people always seek pleasurable experiences that can enhance their well-being when adopting new technology [39,40]. These findings are also consistent with previous results, which showed that an individual’s perceived well-being toward technology will positively influence their perceived ease of use, usefulness, and intention [32,33,63,64].

Furthermore, this study also revealed that older adults’ trust positively influences the perceived usefulness and attitude of telehealth. This finding is predictable because most older adults prefer to receive services in-person and may have trust issues with telehealth’s ability to replace in-person consultations and physical health assessments, so they may perceive high risks and lower trust toward telehealth. The study validates the findings of Rajak and Shaw [46], who revealed the crucial role of trust in the health care technology adoption among older adults.

### Conclusions and Implications

Given the rapid growth of the older adult health care industry, improving health care–related services is a promising opportunity. Hence, this study was conducted to investigate the factors influencing the intention of older adults to use telehealth services by extending the TAM model. The model has been stretched to incorporate factors like availability, transition cost, trust, inertia, and well-being. From the literature, it was observed that a considerable number of studies have been conducted on telehealth. However, there is no evidence in the literature of incorporating these factors and further assessing the model in the Malaysian context. In addition, although several papers have adopted the TAM to investigate telehealth adoption, there are limited studies investigating the formation of the TAM key constructs (perceived ease of use and perceived usefulness) in depth from the older adults’ perspective. With the introduction of these constructs into the TAM model, this study can contribute to the literature by providing a better understanding of factors affecting older adults’ intention to use telehealth in the Malaysian context.

This study has several practical implications. First, the positive impact of attitude toward older adults’ intention on telehealth use provides valuable implications to the health care centers and managers. They should create an environment that can ensure older adults have a positive attitude toward telehealth. On the basis of the findings, positive attitude toward telehealth can be enhanced in several ways, for example, when the older adults perceive it to be useful and easy to use. Hence, the telehealth developer and health care personnel should carefully assess telehealth’s usefulness in assisting older adults’ health care before it is introduced to them.

In addition, telehealth developers should also design an older adult–friendly interface that is easier for them to navigate and understand, as the older adults are not as digitally literate as the younger generation. For example, a case study by Pires et al [96] emphasizes the importance of simplicity and ease of use in successfully implementing the VITASENIOR-MT telehealth system for older adult users. By using television as the primary interface, the telehealth system became more accessible and easier by integrating familiar technology into the homes of older adults. Health professionals provided valuable feedback on design and usability, enhancing the system’s effectiveness as they remotely monitored patients. This case exemplifies the best practices in telehealth design, highlighting the significance of user-centered development that prioritizes ease of use for older adults. In addition, health professionals are encouraged to actively engage in the development process to further improve the system’s effectiveness. A clear step-by-step video tutorial, easy interactive dialogue, and straightforward click-through procedures for making health care appointments can be introduced. Moreover, the government or relevant authorities can provide older adults with training on how to use telehealth services to improve their skills and confidence in accessing online services.

The results from this study revealed that if the transition cost required when using telehealth is perceived as high, it will cause people to have a negative attitude and intention toward telehealth. Furthermore, results also indicate that older adults tend to keep using their habitual ways to obtain health care services, which is known as inertia in this study. This inertia negatively affects their perceived ease of use and usefulness of telehealth. The rationale behind these results might be related to the fact that most commonly available smartphone devices, such as tablet computers and smartphones, as well as mobile internet subscription plans, are generally sold at a high price. Therefore, older adult mobile users only seek to access the internet through free Wi-Fi networks due to the high cost of using mobile devices. Given these considerations, it is recommended that telecommunications companies collaborate with government agencies to launch programs that offer special low prices, rebates, or government-funded subsidies for older adults when they purchase a mobile device and sign up for mobile internet packages. For instance, telecommunication companies in Taiwan have effectively held a promotional campaign for older adults to access the internet for free for a certain period [97]. Through a short, free trial, older adults’ increasing internet accessibility is an effective way to encourage them to learn and become familiar with mobile apps and to reduce their inertia by providing a greater understanding of the mobile services’ value and usefulness, particularly the telehealth services.

In addition to utilitarian-oriented benefits like usefulness and ease of use, which have been frequently discussed in earlier literature, policy makers and managers must also highlight the affective components like well-being [33]. Surprisingly, the result revealed that well-being is the most important factor that affects older adults’ intention for telehealth use. Hence, practitioners should pay more attention to developing a sense of well-being enhancement if older adults use telehealth services. For instance, relevant authorities can create an online community of innovative users where the older adults can share their pleasant, positive, and healthy experiences to highlight the well-being provided by telehealth, and hence indirectly generate positive word of mouth that motivates the potential users to use telehealth. On the other hand, findings also revealed that availability positively influences older adults’ perceived ease of use and usefulness of telehealth. Therefore, technology developers, manufacturers, or organizations should always be prepared to respond and assist the older adult in overcoming challenges and obstacles when using telehealth services, particularly during the early stages of adoption.

Furthermore, the study’s findings also revealed that older adults’ trust positively affects the perceived usefulness. Incorporating trust as a key determinant of perceived usefulness is particularly important for older adults adopting telehealth. The literature suggests that trust in telehealth can be fostered through mechanisms, such as confidence in the competence of health care providers [17,81,82] and the reliability of the information provided [98]. In practice, trust can be strengthened by implementing ongoing professional development and telehealth-specific training for health care providers. This can ensure they remain updated on best practices and patient care, thus boosting patient confidence in their competence. In addition, regularly updating health information and cross-referencing it with evidence-based guidelines reinforces the reliability of the information provided on telehealth platforms.

Moreover, government authorities and telehealth platform developers play a critical role in improving trust in using telehealth services. As a result, it is suggested that government agencies implement clear and detailed support policies for telehealth services. For instance, the government should strengthen the laws to ensure that the platform developers ensure the confidentiality of patient data and data security while health care institutions and staff are qualified. The introduction of national policies shows the government’s support for telehealth services, which could increase the public’s trust toward them [99]. In addition, managers should assist in the development of telehealth platforms by implementing and promoting features and guidelines on patient data confidentiality and privacy protection to establish customer trust. Furthermore, the use of secondary data and security policies should be communicated and advertised by companies to increase trust. In addition, the government should also provide sufficient budget allocation and training for health care professionals to use telehealth services so they can provide better telehealth services for patients and, in turn, increase their trust in telehealth services.

### Limitations and Suggestions for Future Research

Like all research endeavors, this study is not immune to limitations. The first constraint becomes evident through the limited number of determinants examined concerning the intention to use telehealth services. We recognize that numerous potential factors remain unexplored, particularly within the realm of customization.

Second, our research adopts a cross-sectional survey design approach. Given the rapid evolution of technology and the increasing familiarity of consumers with telehealth, this approach may impact their evolving perceptions of telehealth. In addition, consumers’ lifestyles are dynamic, undergoing continuous changes across different life stages. Consequently, a longitudinal study would yield valuable insights into the sustained use of telehealth services. In addition, future research could explore how different types of telehealth services (eg, video consultations vs remote monitoring) specifically impact adoption rates among older adults.

This study used convenient and snowball sampling to reach a hard-to-access population. Although the data represent various ethnicities and regions across Malaysia, the uneven distribution of responses may still introduce a certain level of bias. To enhance representativeness, we recommend using random or stratified sampling methods in future research.

Another limitation is the potential for self-report biases, as participants may have provided socially desirable responses rather than their true thoughts or behaviors. While we mitigated this by ensuring survey anonymity, self-report biases may still persist and should be considered when interpreting the findings. Future research should incorporate objective measures, such as tracking actual telehealth use data, to complement self-reported data and provide a more accurate assessment.

Although this study meets the sample size requirements based on power analysis using G-Power, it may not fully capture the diversity of the older adult population, potentially limiting the generalizability of the findings. Caution is advised when applying these results to the broader older adult population. Future studies should include larger, more diverse samples across different socioeconomic backgrounds, geographic locations, and health conditions to strengthen the robustness and applicability of the findings.

## Appendix

Multimedia Appendix 1 Overview of the instrument.

Multimedia Appendix 2 Respondents’ information (N=119).

Multimedia Appendix 3 Bias-corrected interval, f2, and variance inflation factor.

## Abbreviations

HTMT: heterotrait-monotrait
IPMA: importance-performance map analysis
PLS-SEM: partial least squares structural equation modeling
TAM: technology acceptance model
