# Supplementary material for: Determinants of Telehealth Adoption Among Older Adults: Cross-Sectional Survey Study
Source: JMIR Aging. 2025 Mar 24;8:e60936. doi: 10.2196/60936 (PMC11976177; doi:10.2196/60936)
Supplement: Multimedia Appendix 1 [file aging_v8i1e60936_app1.docx]

Multimedia Appendix 1. Overview of the instrument.

| Constructs | Items | Reference |
| --- | --- | --- |
| Attitude | - Using telehealth is a good idea. | [36,83] |
|  | - Telehealth is easier to work than traditional healthcare practices. |  |
|  | - I would enjoy using telehealth. |  |
| Perceived ease of use | - It is easy to learn how to apply telehealth. | [36,83] |
|  | - It is easy to use telehealth. |  |
|  | - In general, it is easy to apply telehealth. |  |
| Perceived usefulness | - Using telehealth would improve my current health condition. | [36,83] |
|  | - Using telehealth helps me to do my health-related activities easily. |  |
|  | - Telehealth is very useful to my life in general. |  |
|  | - Telehealth provides a very useful service and information to me. |  |
| Transition Cost | - I do not want to use telehealth because it will be a problem to install and operate the new technology. | [36,83] |
|  | - It will take me a lot of time and effort to switch to telehealth. |  |
|  | - In general, it is troublesome to switch to telehealth. |  |
| Inertia | - I will continue to use offline medical services because they are part of my life. | [36] |
|  | - Even though offline medical services do not have good effectiveness, I will continue to use them. |  |
|  | - I am already used to these offline medical services. |  |
| Availability | - Applying telehealth allows me to receive information rapidly, such as information about health and medication. | [36] |
|  | - I can receive information via telehealth at any time and in any place, such as information about health and medications. |  |
|  | - Information provided by telehealth updates regularly. |  |
| Trust | - Doctors can provide telehealth service well. | [84] |
|  | - The information provided through telehealth is valid. |  |
|  | - The people I interact with through telehealth platforms are trustworthy. |  |
| Intention | - I am willing to use telehealth. | [83,84] |
|  | - I will try to replace my current physical measurement tools with telehealth. |  |
|  | - I plan to use telehealth. |  |
| Subjective Well Being | - I believe after I used the telehealth, my life close to my ideal. | [32] |
|  | - I believe after I used the telehealth, the condition of my life become excellent. |  |
|  | - I believe after I used the telehealth, I am satisfied with my life. |  |

References

32. Yap Y-Y, Tan S-H, Tan S-K, Choon S-W. Integrating the capability approach and technology acceptance model to explain the elderly’s use intention of online grocery shopping. Telemat Informatics 2022 Aug;72:101842. doi: [10.1016/j.tele.2022.101842](https://doi.org/10.1016/j.tele.2022.101842)

34. Tsai J-M, Cheng M-J, Tsai H-H, Hung S-W, Chen Y-L. Acceptance and resistance of telehealth: The perspective of dual-factor concepts in technology adoption. Int J Inf Manage 2019 Dec;49:34–44. doi: [10.1016/j.ijinfomgt.2019.03.003](https://doi.org/10.1016/j.ijinfomgt.2019.03.003)

83. Zhang X, Zaman B uz. Adoption mechanism of telemedicine in underdeveloped country. Health Informatics J 2020 Jun 30;26(2):1088–1103. doi: [10.1177/1460458219868353](https://doi.org/10.1177/1460458219868353)

84. Wu D, Gu H, Gu S, You H. Individual motivation and social influence: a study of telemedicine adoption in China based on social cognitive theory. Heal Policy Technol 2021 Sep;10(3):100525. doi: [10.1016/j.hlpt.2021.100525](https://doi.org/10.1016/j.hlpt.2021.100525)
