# Supplementary material for: Determinants of Telehealth Adoption Among Older Adults: Cross-Sectional Survey Study
Source: JMIR Aging. 2025 Mar 24;8:e60936. doi: 10.2196/60936 (PMC11976177; doi:10.2196/60936)
Supplement: Multimedia Appendix 2 [file aging_v8i1e60936_app2.docx]

Multimedia Appendix 2. Respondents’ information (N = 119).

| Characteristics | | Participants |
| --- | --- | --- |
| **Gender, n (%)** | |  |
|  | Female | 52 (43.7) |
|  | Male | 67 (56.3) |
| **Ethnicity, n (%)** | |  |
|  | Malay | 45 (37.8) |
|  | Chinese | 56 (47.1) |
|  | Indian | 15 (12.6) |
|  | Others | 3 (2.5) |
| **Region, n (%)** | |  |
|  | Northern | 16 (13.4) |
|  | Central | 62 (52.1) |
|  | Southern | 21 (17.6) |
|  | East Coast Region | 17 (14.3) |
|  | Eastern Malaysia | 3 (2.5) |
