# Supplementary material for: Determinants of Telehealth Adoption Among Older Adults: Cross-Sectional Survey Study
Source: JMIR Aging. 2025 Mar 24;8:e60936. doi: 10.2196/60936 (PMC11976177; doi:10.2196/60936)
Supplement: Multimedia Appendix 3 [file aging_v8i1e60936_app3.docx]

| Hypothesis | Relationship | BCI LL | BCI UL | f^2^ | VIF |
| --- | --- | --- | --- | --- | --- |
| H1 | Attitude🡪 Intention | 0.140 | 0.331 | 0.074 | 2.250 |
| H2a | Perceived ease of use 🡪 Perceived usefulness | 0.195 | 0.353 | 0.111 | 1.939 |
| H2b | Perceived ease of use 🡪 Attitude | 0.117 | 0.302 | 0.041 | 2.160 |
| H3a | Perceived usefulness 🡪 Attitude | 0.363 | 0.539 | 0.230 | 1.807 |
| H3b | Perceived usefulness 🡪 Intention | 0.021 | 0.186 | 0.013 | 2.148 |
| H4a | Transition cost 🡪 Attitude | -0.245 | -0.065 | 0.031 | 1.535 |
| H4b | Transition cost 🡪 Intention | -0.224 | -0.102 | 0.055 | 1.380 |
| H**5**a | Subjective well-being 🡪 Perceived ease of use | 0.171 | 0.346 | 0.090 | 1.437 |
| H**5**b | Subjective well-being 🡪 Perceived usefulness | 0.165 | 0.353 | 0.088 | 2.192 |
| H**5**c | Subjective well-being 🡪 Intention | 0.374 | 0.528 | 0.269 | 2.138 |
| H**6**a | Inertia 🡪 Perceived ease of use | -0.308 | -0.173 | 0.100 | 1.129 |
| H**6**b | Inertia 🡪 Perceived usefulness | -0.027 | 0.083 | 0.002 | 1.252 |
| H**7**a | Availability 🡪 Ease of use | 0.342 | 0.499 | 0.261 | 1.293 |
| H**7**b | Availability 🡪 Perceived usefulness | 0.245 | 0.400 | 0.182 | 1.647 |
| H**8** | Trust 🡪 Perceived usefulness | 0.062 | 0.262 | 0.038 | 1.903 |

Multimedia Appendix 3. Biased Corrected Interval, f^2^ and Variance Inflation Factor (VIF).
